# Identification of mineralocorticoid receptor target genes in the mouse hippocampus

**DOI:** 10.1111/jne.12735

**Published:** 2019-06-14

**Authors:** Lisa T. C. M. van Weert, Jacobus C. Buurstede, Hetty C. M. Sips, Sabine Vettorazzi, Isabel M. Mol, Jakob Hartmann, Stefan Prekovic, Wilbert Zwart, Mathias V. Schmidt, Benno Roozendaal, Jan P. Tuckermann, R. Angela Sarabdjitsingh, Onno C. Meijer

**Affiliations:** ^1^ Einthoven Laboratory Division of Endocrinology Department of Medicine Leiden University Medical Center Leiden The Netherlands; ^2^ Department of Cognitive Neuroscience Radboud University Medical Center Nijmegen The Netherlands; ^3^ Donders Institute for Brain, Cognition and Behaviour Radboud University Nijmegen The Netherlands; ^4^ Institute of Comparative Molecular Endocrinology University of Ulm Ulm Germany; ^5^ Department of Psychiatry Harvard Medical School McLean Hospital Belmont Massachusetts; ^6^ Division of Oncogenomics Oncode Institute The Netherlands Cancer Institute Amsterdam The Netherlands; ^7^ Department of Stress Neurobiology and Neurogenetics Max Planck Institute of Psychiatry Munich Germany; ^8^ Department of Translational Neuroscience UMC Utrecht Brain Center University Medical Center Utrecht The Netherlands

**Keywords:** glucocorticoids, *Jdp2*, mineralocorticoid receptor knockout, restraint stress, transcription

## Abstract

Brain mineralocorticoid receptors (MRs) and glucocorticoid receptors (GRs) respond to the same glucocorticoid hormones but can have differential effects on cellular function. Several lines of evidence suggest that MR‐specific target genes must exist and might underlie the distinct effects of the receptors. The present study aimed to identify MR‐specific target genes in the hippocampus, a brain region where MR and GR are co‐localised and play a role in the stress response. Using genome‐wide binding of both receptor types, we previously identified MR‐specific, MR‐GR overlapping and GR‐specific putative target genes. We now report altered gene expression levels of such genes in the hippocampus of forebrain MR knockout (fbMRKO) mice, killed at the time of their endogenous corticosterone peak. Of those genes associated with MR‐specific binding, the most robust effect was a 50% reduction in *Jun dimerization protein 2* (*Jdp2*) mRNA levels in fbMRKO mice. Down‐regulation was also observed for the MR‐specific *Nitric oxide synthase 1 adaptor protein* (*Nos1ap*) and *Suv3 like RNA helicase* (*Supv3 l1*). Interestingly, the classical glucocorticoid target gene *FK506 binding protein 5* (*Fkbp5*), which is associated with MR and GR chromatin binding, was expressed at substantially lower levels in fbMRKO mice. Subsequently, hippocampal *Jdp2* was confirmed to be up‐regulated in a restraint stress model, posing *Jdp2* as a bona fide MR target that is also responsive in an acute stress condition. Thus, we show that MR‐selective DNA binding can reveal functional regulation of genes and further identify distinct MR‐specific effector pathways.

## 1 INTRODUCTION

Endogenous glucocorticoid hormones affect brain function via two closely‐related nuclear receptors: the mineralocorticoid receptor (MR) and the glucocorticoid receptor (GR). The ligand concentration in part determines the specific MR/GR responses. High affinity MRs are occupied by endogenous corticosteroids under basal conditions and have been found to be more relevant in the initial phase of a stress response.^1^, ^2^ By contrast, the lower affinity GRs are activated only at higher glucocorticoid levels, around the peak of the circadian rhythm and during a stress response. Although GRs are expressed widely throughout the central nervous system, brain glucocorticoid binding MRs are mainly restricted to limbic areas.^3^

In the hippocampus, MR and GR are crucial for spatial memory and the modulation of cognition, mood and behaviour.^3^ Within the CA1 hippocampal subregion, MR and GR mediate opposite glucocorticoid effects on pyramidal neurone excitability^4^ via transcriptional mechanisms.^5^ Also, spatial learning in rodents is differentially affected by MR and GR signalling, with MR modulating response selection and GR being essential for memory consolidation.^6^, ^7^ Because of intrinsic MR‐mediated effects that oppose those of GR, it has long been argued that MR‐specific target genes must exist.^8^ The existence of MR‐specific transcriptional coregulators^9^, ^10^ also supports this idea. However, many of the effects that can be attributed specifically to MR function so far comprise rapid non‐genomic effects, mediated by the membrane variant of the receptor.^11^, ^12^

Several classical genomic MR‐targets have been described in various tissues over the past two decades, such as *FK506 binding protein 5* (*Fkbp5*),^13^ *glucocorticoid‐induced leucine zipper* (*Gilz*),^14^ *period circadian clock 1* (*Per1*)^15^ and *serum/glucocorticoid regulated kinase 1* (*Sgk1*).^16^ However, these genes are all known to be GR responsive as well.^17^, ^18^, ^19^, ^20^ Of note, not only can the two receptors bind their target DNA as homodimers, but also heterodimerisation of MR/GR has been described.^15^ Although MR‐selective transrepression and transactivation may occur,^21^, ^22^ to date, no hippocampal genomic targets have been reported that are strictly MR‐dependent. Transcriptional changes have been attributed to MR function,^23^ although they were not formally proven to be direct targets of the receptor and might thus be affected by MR activity in an indirect manner. However, although the presence of glucocorticoid response element (GRE) appears to be crucial for both MR and GR DNA binding in the hippocampus, binding sites for NeuroD transcription factors were found selectively at MR‐bound loci.^24^ NeuroD factors could coactivate glucocorticoid‐induced transactivation and were indeed present near MR‐specific binding sites, suggesting that specific GRE‐dependent MR target genes do exist.

The present study investigated whether direct MR binding to the hippocampal DNA led to expression regulation of the nearby gene. Based on our recent work that defined MR‐specific, MR‐GR overlapping and GR‐specific chromatin binding sites and the corresponding putative target genes within the rat hippocampus,^24^ we examined mRNA levels of several genes in each of these categories. Forebrain MR knockout (fbMRKO) mice showed altered expression for a subset of genes, including down‐regulation of the mixed MR/GR target *Fkbp5*, as well as MR‐specific *Jun dimerization protein 2* (*Jdp2*), *Nitric oxide synthase 1 adaptor protein* (*Nos1ap*) and *Suv3 like RNA helicase* (*Supv3 l1*) mRNA levels. Subsequently, corticosterone responsiveness of *Jdp2*, which is one of the genes with an MR‐bound promoter, was validated in mice that were exposed to different durations of restraint stress.

## 2 MATERIALS AND METHODS

### 2.1 Animals

Male homozygous fbMRKO and control c57bl/6 mice (n = 7) aged 8‐9 weeks were housed under a 12:12 hour light/dark reversed photocycle (lights off 9.00 am). The fbMRKO mice were generated using MR^flox^ mice, with MR exon 3 flanked by loxP sites, and mice expressing Cre recombinase controlled by the CAMKIIα gene.^25^ Male MR^flox/flox^CamKCre^Cre/wt^ mice were crossed with female MR^flox/flox^ mice to generate fbMRKO (MR^flox/flox_Cre^) and control (MR^flox/flox_wt^) offspring. Because the breeding unexpectedly generated more fbMRKO than control mice, only part of the control animals were littermates. No differences were found in expression levels between littermate and non‐littermate controls for any of the genes measured. Mice were transferred to a novel cage 20 minutes before harvesting the tissue and then killed around the time of their endogenous corticosterone peak, between 9.30 am and 12.00 pm. We assessed the expression of MR, overlapping and GR putative target genes in this condition because both receptor types are activated at peak of the diurnal corticosterone rhythm. The novel cage was included in the protocol to ensure MR and GR binding for chromatin immunoprecipitation (ChIP) analysis in the same animals, under the assumption that mRNA levels will not be affected in this short time span. Trunk blood was collected from all mice and hippocampal hemispheres were freshly dissected and snap‐frozen in liquid nitrogen for later analysis.

For validation of *Jdp2* down‐regulation, male fbMRKO (n = 14) and littermate controls (n = 10) aged 8‐12 weeks were housed under a 12:12 hour light/dark photocycle (lights on 7.00 am). Mice were bred as described above. Mice were killed under baseline conditions, between 9.30 am and 10.30 am. Brains were collected and snap‐frozen in liquid nitrogen for later analysis.

For MR binding site validation in the mouse brain, male c57bl/6 mice (n = 5) aged 16‐19 weeks were housed under a 12:12 hour light/dark photocycle (lights on 8.00 am) and were killed in the afternoon 60 minutes after an i.p. injection of 3.0 mg kg^‐1^ corticosterone (Sigma, St Louis, MO, USA) dissolved in 5% ethanol in saline, ensuring MR binding. Hippocampal hemispheres were freshly dissected and snap‐frozen in liquid nitrogen for later analysis.

Male Balb/c mice (n = 3‐6) aged 8‐15 weeks were housed under a 12:12 hour light/dark photocycle (lights on 6.00 am) and were exposed to various periods of restraint stress (0, 30, 60, 120 and 240 minutes) and killed directly afterwards, between 9.30 am and 2.00 pm. At this time of the diurnal corticosterone trough, both MR and GR DNA binding can be enhanced in response to stress^15^ and consequential gene expression changes compared to non‐stressed control mice could be revealed. Trunk blood was collected from all mice, and hippocampal hemispheres were freshly dissected and snap‐frozen in liquid nitrogen for later analysis.

All experiments were performed in accordance with the European Commission Council Directive 2010/63/EU and the Dutch law on animal experiments and approved by the animal ethical committee from Utrecht University, University of Amsterdam, or the German Regierungspräsidium Tübingen.

### 2.2 Plasma measurements

Trunk blood was centrifuged for 10 minutes at 7000 *g*, after which plasma was transferred to new tubes. Corticosterone levels of the fbMRKO experiment were determined using an enzyme immunoassay (Immunodiagnostic Systems, East Boldon, UK) and adrenocorticotrophic hormone (ACTH) and corticosterone levels of the restraint stress mice were determined using an enzyme‐linked immunosorbent assay (IBL International, Hamburg, Germany) in accordance with the manufacturers’ instructions.

### 2.3 Target gene selection

MR‐specific, MR‐GR overlapping and GR‐specific binding sites were annotated to the nearest gene.^24^ To increase the chances of correct annotation and identifying functional target genes, we focused on binding sites located intragenic or in the proximal promoter (up to‐5 kb). Furthermore, hippocampal expression^26^ of the putative target genes, the degree of coexpression with NeuroD factors (Neurod1/2/6) and face validity of ChiP‐sequencing (ChIP‐seq) peaks were assessed. The total numbers of putative target genes measured for MR‐specific, overlapping (including classical targets) and GR‐specific subset were 12, 10 and 9, respectively.

### 2.4 ChIP‐quantitative polymerase chain reaction (PCR)

For MR binding validation in the mouse, we performed ChIP‐quantitative PCR on hippocampal tissue of wild‐type (WT) mice (n = 5) as described previously.^27^ Hippocampal hemispheres were cryosectioned at 30 μm before cross‐linking with 2 mmol L^‐1^ disuccinimidyl glutarate, followed by 1% formaldehyde. Fixated tissue was suspended, nuclei were isolated and sonicated for 10 rounds (30 seconds ON/30 seconds OFF) using a Bioruptor Pico (Diagenode, Seraing, Belgium). Chromatin of two hemispheres of the same animal was pooled and used for a single ChIP sample (500 μL) to measure MR binding with 5 μg of anti‐MR antibody (21854‐1‐AP; ProteinTech, Rosemont, IL, USA). Immunoprecipitation was performed with 50 μL of magnetic Protein A beads (Dynabeads; Invitrogen, Carlsbad, CA, USA). The background signal was detected for each sample with a sequential ChIP using 5 μg of control immunoglobulin (Ig)G antibody (ab37415; Abcam, Cambridge, MA, USA). Pellets were dissolved in 50 μL 10 mmol L^‐1^ Tris‐HCl (pH 8). Subsequently, a quantitative PCR was performed on 5 × diluted ChIP samples, with primers that were designed to span the GRE of the MR binding sites (Table 1).

**Table 1:** Primer sequences used for a quantitative polymerase chain reaction in mouse hippocampal chromatin immunoprecipitation samples (for binding site details, see Table 3)

Binding site	Nearest gene	Forward and reverse (5′‐ to 3′)	Product length (bp)
GR3000_1726	*Acsl6*	CCTGCCAGGAGAGCAGATGTGTGCAGGAAGGCAAGTTCT	178
MR3000_740GR3000_34	*Fkbp5*	TGCCAGCCACATTCAGAACATCAAGTGAGTCTGGTCACTGC	122
MR3000_1054	*Jdp2*	AAGTAAGACCGCGACCTACAAAATACCCAGTGCAGAGACGAA	192
MR300_473GR3000_599	*Kif1c*	GCTGGGGTGTACACAGATGGTGACTAGCCAGAGCAGTATGTC	156
GR3000_106	*Mrpl48*	AGCTGTGCTTTGGAAGCCTACATAAGGTGGGCCACACTCC	170
MR300_196	*Nos1ap*	CCTCCGATGCTGCTTGGATACAGACCGAGCCAGCGATAAG	197
MR3000_738GR3000_12	*Per1*	GGAGGCGCCAAGGCTGAGTGCGGCCAGCGCACTAGGGAAC	73
MR300_503	*Rilpl1*	CAGGCAGATGCCAGGCTCCCATGCCTGTTCCTCTAGT	106
MR3000_359	*Supv3 l1*	TGCAGGGATTCGATGGACAGCTCTGAGCCACCTCTCAAGC	165
MR3000_641GR3000_1603	*Zfp219*	AGTCCATCACATTCTGTTGCTTTCTAGTCAGCTATGACCATGCAGT	131

### 2.5 Real‐time quantitative PCR

Mouse hippocampal hemispheres were homogenised in TriPure (Roche, Basel, Switzerland) by shaking the tissue with 1.0‐mm diameter glass beads for 20 seconds at 6.5 m s^‐1^ in a FastPrep‐24 5G instrument (MP Biomedicals, Santa Ana, CA, USA). Total RNA was isolated, cDNA was generated and quantitative real‐time PCR was performed as described previously.^24^ Because *Actb* (β‐actin) expression was regulated between fbMRKO and control mice, genes of interest were normalised against the in both experiments stably expressed housekeeping gene *Rplp0*, encoding a ribosomal protein. Primer sequences are listed in Table 2.

**Table 2:** Primer sequences used for a quantitative real‐time polymerase chain reaction in the mouse hippocampus

Gene	Full name	Forward and reverse (5′‐ to 3′)	Product length (bp)
*Acsl6*	Acyl‐CoA synthetase long‐chain family member 6	TCTCAGGGAATGGACCCTGTCCTCTTGGTAGGACAGCCAC	135
*Bhlhb9*	Basic helix‐loop‐helix domain containing, class B9	AACTCACCTGGCCAGCAATCCTCTGGCTGCCTTGGGATTT	187
*C4ST1 (Chst11)*	Chondroitin 4‐sulfotransferase 1	GAATTTGCCGGATGGTGCTGAGCAGATGTCCACACCGAAG	117
*Camk1d*	Calcium/calmodulin‐dependent protein kinase ID	GCATCGAGAACGAGATTGCCCCAGACACAAGTTGCATGACC	114
*Camkk2*	Calcium/calmodulin‐dependent protein kinase kinase 2	AGAACTGCACACTGGTCGAGACCAGGATCACAGTTGCCAG	85
*Fkbp5*	FK506 binding protein 5	TCCTGGGAGATGGACACCAATTCCCGTACTGAATCACGGC	113
*Gilz (Tsc22d3)*	Glucocorticoid‐induced leucine zipper	TGGCCCTAGACAACAAGATTGAGCCCACCTCCTCTCTCACAGCAT	78
*Hsd17b11*	Hydroxysteroid (17‐β) dehydrogenase 11	CGCAGGACCCTCAGATTGAAGGAGCAGTAAGCCAGCAAGA	167
*Jdp2*	Jun dimerization protein 2	TACGCTGACATCCGCAACATCGTCTAGCTCACTCTTCACGG	100
*Kif1c*	Kinesin family member 1C	TTAATGCCCGTGAGACCAGCAAGCTTTTGGGGGCATCCTT	106
*Mrpl48*	Mitochondrial ribosomal protein L48	CAGTATGTCCACCGCCTCTGCTCGCTCATGGGTGGTAAGG	145
*Nos1ap*	Nitric oxide synthase 1 adaptor protein	TGGAATTCAGCCGAGGTGTGGGAAGGGAGCAGCATTCGAG	131
*Nr3c1* (GR)	Nuclear receptor subfamily 3, group C, member 1	CCCTCCCATCTAACCATCCTT ACATAAGCGCCACCTTTCTG	89
*Nr3c2* (MR)	Nuclear receptor subfamily 3, group C, member 2	TCCAAGATCTGCTTGGTGTGCCCAGCTTCTTTGACTTTCG	239
*Per1*	Period circadian clock 1	ACGGCCAGGTGTCGTGATTACCCTTCTAGGGGACCACTCA	162
*Rilpl1*	Rab interacting lysosomal protein‐like 1	ACGAGCTCAAGTCCAAGGTGAGTCGCTTGATCCCCGATTC	148
*Rplp0*	Ribosomal protein, large, P0	GGACCCGAGAAGACCTCCTTGCACATCACTCAGAATTTCAATGG	85
*Sgk1*	Serum/glucocorticoid regulated kinase 1	AGAGGCTGGGTGCCAAGGATCACTGGGCCCGCTCACATTT	129
*Supv3 l1*	Suv3 like RNA helicase	CTCACTCGGCCTCTAGACAAGTCCACGTCCAGAGAATGGGA	170
*Zfp219*	Zinc finger protein 219	GATCTGCAGCGCTACTCCAATGCACGAGTCTCAGACCAAC	96

### 2.6 In situ hybridisation

Frozen brains were sectioned at 18 μm in a cryostat microtome, collected on SuperFrost Plus slides (Thermo Scientific, Walthaqm, MA, USA) and stored at −80°C until further use. In situ hybridisation using ^35^S UTP‐labelled ribonucleotide probes for *Jdp2* was performed as described previously.^28^

### 2.7 Statistical analysis

In the fbMRKO experiment, independent *t*‐tests were used and *P *<* *0.01 was considered as a statistically significance cut‐off to correct for multiple gene testing. For the ChIP‐quantitative PCR validation, we performed one‐tailed paired *t* tests. The predictable directionality (ie, the MR signal is higher than the background IgG signal) justifies the use of a one‐tailed test. Because a decrease in signal might also be relevant, we note that all statisitically significant results were at *P* < 0.025 and therefore would also be significant using a two‐tailed test. We considered a paired test appropriate because MR and IgG are measured on the same chromatin sample and this allows correction for the corresponding background levels. Again, one‐tailed unpaired *t* tests gave essentially the same results. For the gene *Nos1ap*, one of the samples was excluded from analysis because of a missing value as a result of non‐detectable IgG levels. For the time course of restraint stress, a one‐way ANOVA was performed with Holm–Sidak's multiple comparison post‐hoc tests. In the in situ measurements of the fbMRKO animals, unpaired *t* tests were performed. *P *<* *0.05 was considered statistically significantly significant unless stated otherwise. prism, version 7 (GraphPad Software Inc., San Diego, CA, USA) was used to analyse the data. All graphs show individual values and data are further depicted as the mean ± SEM.

## 3 RESULTS

**Figure 1:**
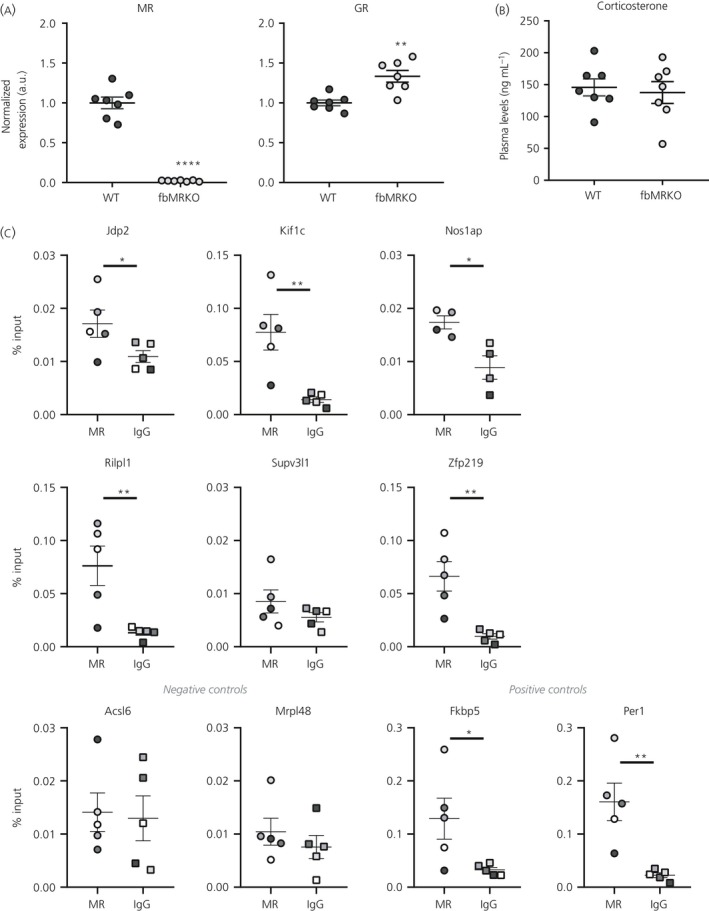
Validation of mineralocorticoid receptor (MR) detection in wild‐type (WT) mice and absence of MR in forebrain MR knockout (fbMRKO) mice. (A) Hippocampal mRNA levels showing MR down‐regulation and slight GR up‐regulation, as well as (B) unaltered plasma corticosterone levels in fbMRKO vs WT mice, as assessed by independent *t*‐tests. (C) MR binding assessed by a chromatin immunoprecipitation‐quantitative polymerase chain reaction in the hippocampus of WT mice, along with an immunoglobulin (Ig)G background signal per sample, as assessed by one‐tailed paired *t*‐tests. Corresponding measurements are depicted in the same colour. GR‐specific targets *Acsl6* and *Mrpl48* served as negative controls; classical glucocorticoid targets *Fkbp5* and *Per1* served as positive controls. a.u., arbitrary unit. *P <* *0.05, **P <* *0.01, ****P <* *0.0001

To explore the functional effects of previously detected MR/GR DNA binding (ie, transcription regulation), binding sites were associated with their nearest gene. This resulted in lists of MR‐specific, MR‐GR overlapping and GR‐specific putative target genes.^24^ Gene expression levels, for a subset of each category (Table 3), were measured in fbMRKO mice at the time of their diurnal corticosterone peak. MR mRNA expression was indeed abolished and GR mRNA was slightly up‐regulated in the hippocampus of fbMRKO mice (Figure 1A), confirming earlier reports.^25^ MR protein levels also showed efficient knockdown Bonapersona V., Damsteegt R., Adams M.L., van Weert L., Meijer O.C., Joëls M., Sarabdjitsingh R.A.(unpublished). Furthermore, no differences were found in plasma corticosterone levels of these animals at the time of death (Figure 1B). Because the studied target loci were originally detected in the rat brain,^24^ we validated MR binding in mice. ChIP‐quantitative PCR confirmed hippocampal MR binding at the *Jdp2* (*P *=* *0.0124), *Kif1c* (*P *=* *0.0087), *Nos1ap* (*P *=* *0.0172), *Rilpl1* (*P *=* *0.0098) and *Zfp219* (*P *=* *0.0049) loci in WT mice, although this signal did not exceed background IgG levels at the GR‐specific sites near *Acsl6* (*P *=* *0.4410) and *Mrpl48* (*P *=* *0.2142) (Figure 1C). Only for *Supv3 l1* (*P *=* *0.1784) were we unable to detect the expected MR binding. Also, for classical target genes *Fkbp5* (*P *=* *0.0246) and *Per1* (*P *=* *0.0066), MR enrichment was demonstrated.

**Table 3:**
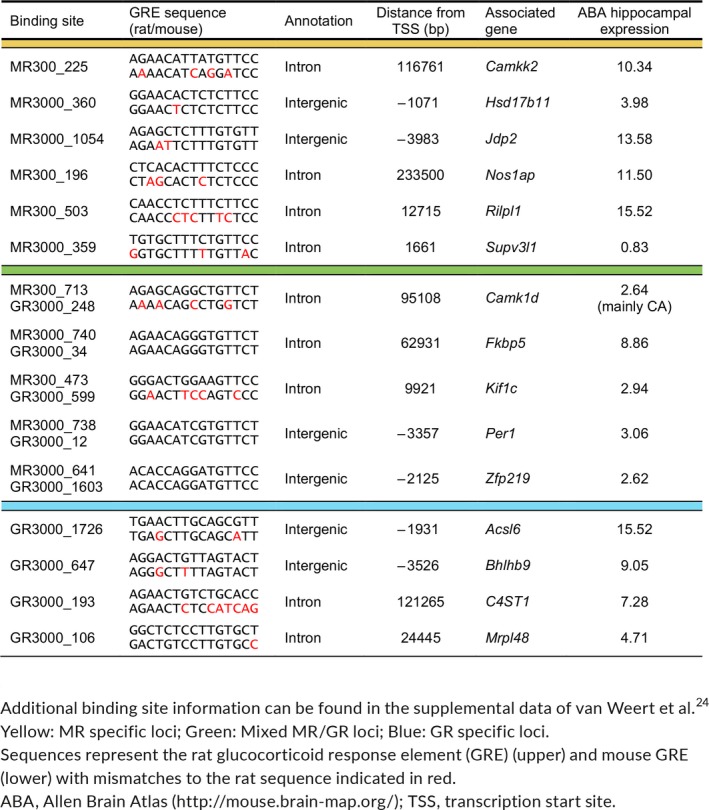
Selected putative target genes to validate

**Figure 2:**
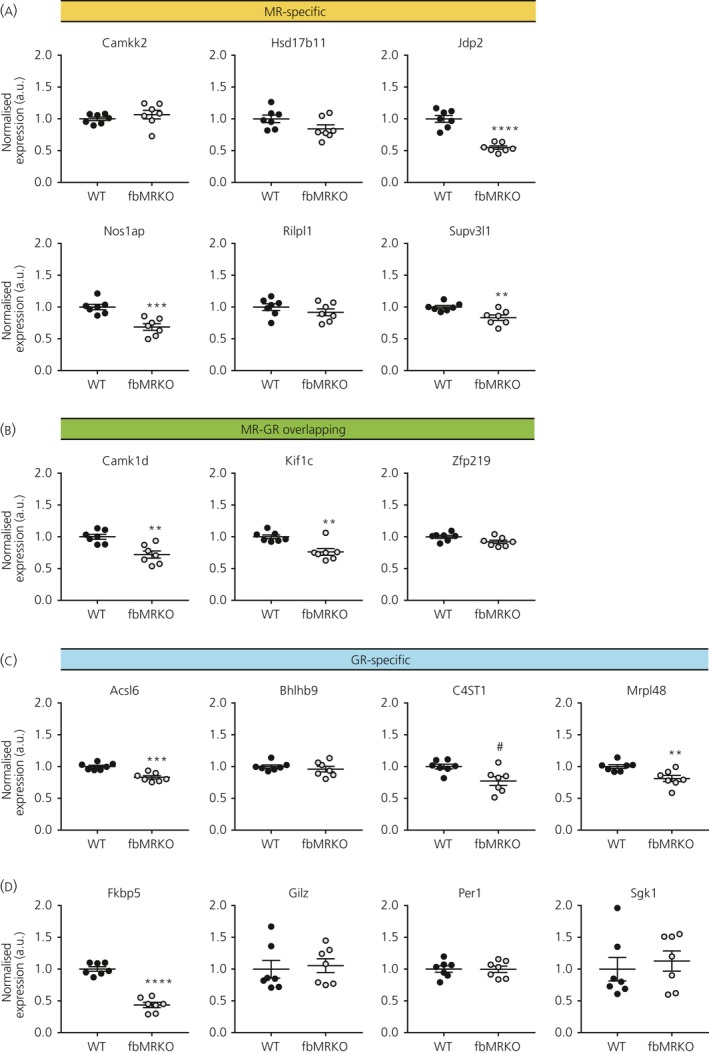
Hippocampal mRNA levels of glucocorticoid target genes assessed in wild‐type (WT) and forebrain mineralocorticoid receptor (MR) knockout (fbMRKO) mice. Gene expression of (A) MR‐specific, (B) overlapping and (C) glucocorticoid receptor (GR)‐specific targets and (D) classical glucocorticoid targets in fbMRKO versus WT mice, as assessed by independent *t*‐tests with *P *<* *0.01 as the statistically significant cut‐off. Other genes measured but not differentially expressed between WT and fbMRKO mice were: *Adam23*,* Arl8b*,* Dgkb*,* Els1*,* Myo16* and *Nob1* as MR‐specific targets; *Grb2*,* Luzp1* and *Map1lc3b* as overlapping targets; *Arntl*,* B3galt1*,* Map2k5*,* Pglyrp1* and *Slc3a2* as GR‐specific targets. a.u., arbitrary unit. #*P *<* *0.05 (considered a trend), **P <* *0.01, ***P <* *0.001, ****P <* *0.0001

Several MR‐specific putative targets showed lower expression levels in the fbMRKO compared to WT mice (Figure 2A). The most robust effect was found in the *Jdp2* mRNA levels, which were reduced by 50% (*P *<* *0.0001). Other differentially expressed genes were MR‐specific *Nos1ap* (*P *=* *0.0005) and *Supv3 l1* (*P *=* *0.0061), as well as MR‐GR overlapping *Camk1d* (*P *=* *0.0016) and *Kif1c* (*P *=* *0.0022), which were also all down‐regulated in the fbMRKO compared to WT mice (Figure 2A,B). Moreover, two of the GR‐specific genes, *Acsl6* (*P *=* *0.0002) and *Mrpl48* (*P *=* *0.0065), were expressed at lower levels, and *C4ST1* showed a trend for lowered expression (*P *=* *0.0138) (Figure 2C).

Besides the brain‐related putative MR/GR target genes, we measured the expression of the classical target genes *Fkbp5*,* Gilz*,* Per1* and *Sgk1* (Figure 2D). These genes are all known to be bound and/or regulated by both MR and GR, comprising our identified MR‐GR overlapping target subset contained *Fkbp5* and *Per1* (Table 3). Of the four classical targets, only *Fkbp5* was down‐regulated in fbMRKO mice, reaching 44% of the levels observed in WT animals (*P *<* *0.0001).

**Figure 3:**
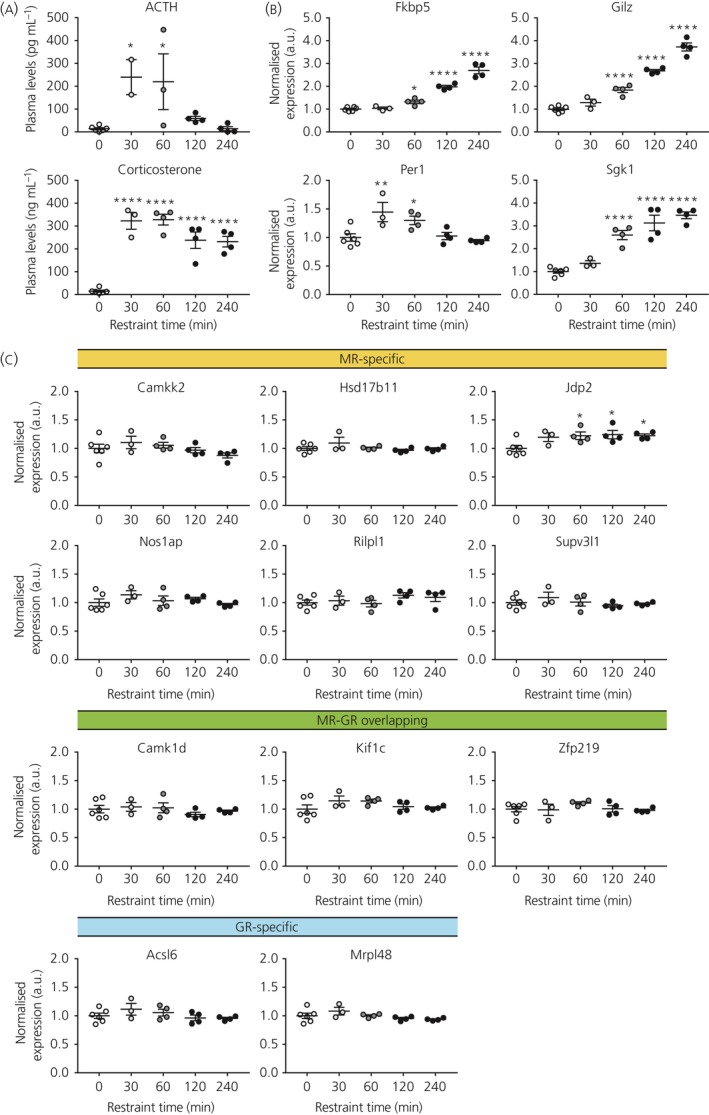
Hippocampal mRNA levels of glucocorticoid target genes assessed in a restraint stress model. A, Plasma adrenocorticotrophic hormone (ACTH) and corticosterone levels after different durations of restraint stress. B, Validation of time‐dependent classical glucocorticoid target gene activation upon restraint stress. C, Gene expression of MR‐specific, overlapping and GR‐specific targets after different durations of restraint stress. All assessed by one‐way ANOVA with Holm‐Sidak's post‐hoc tests. a.u., arbitrary unit. *P <* *0.05, **P <* *0.01, ****P <* *0.0001

Next, we aimed to show regulation of the target genes in an acute stress context. Even though MR is substantially occupied by ligand under basal glucocorticoid levels, MR (and GR) DNA binding and subsequent transcriptional effects can be enhanced by a rise of corticosterone.^15^ Hippocampal gene expression was assessed in mice that were exposed to restraint stress of different durations (0, 30, 60, 120 and 240 minutes). Plasma corticosterone levels were increased after all durations of restraint stress, although they tended to return to baseline at 120 and 240 minutes, in line with the fact that ACTH levels were normalised at these time points (Figure 3A).

Of the classical glucocorticoid target genes, *Fkbp5*,* Gilz* and *Sgk1* were up‐regulated after 60, 120 and 240 minutes of restraint (Figure 3B). *Per1* showed a transient increase, with elevated levels at 30 and 60 minutes, which had declined again from 120 minutes of restraint stress. Interestingly, the MR‐exclusive target gene *Jdp2* that was mostly affected in fbMRKO mice showed an increase in response to stress (Figure 3C) in those animals exposed to restraint for 60‐240 minutes. Other genes associated with MR and/or GR binding loci that we had selected for validation did not show transcriptional effects upon restraint stress (Figure 3C).

**Figure 4:**
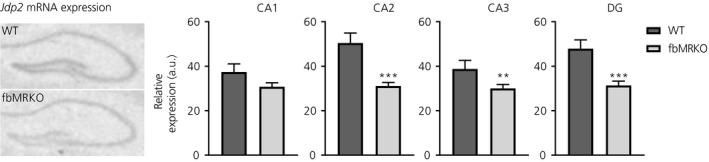
Validation of hippocampal *Jdp2* down‐regulation in forebrain mineralocorticoid receptor (MR) knockout (fbMRKO) mice compared to wild‐type (WT) mice, detected by in situ hybridisation, as assessed by unpaired *t*‐tests. Left: representative scanned autoradiograph film per genotype. Gene expression is quantified per subregion of the hippocampus: cornu ammonis (CA)1, CA2, CA3 and the dentate gyrus (DG). a.u., arbitrary unit. **P <* *0.01, ***P <* *0.001

Finally, we confirmed *Jdp2* down‐regulation measured by in situ hybridisation in an independent experiment in fbMRKO (Figure 4). In the absence of MR, *Jdp2* mRNA levels were decreased in the principal neurones of the dorsal hippocampus, as was apparent from a significantly lower expression in the CA2 (*P *=* *0.0001), CA3 (*P *=* *0.0357) and dentate gyrus (*P *=* *0.0005) subregions. For the CA1, this occurred at the trend level (*P *=* *0.0901).

## 4 DISCUSSION

Based on non‐overlapping MR‐GR binding sites, we defined putative MR‐specific and GR‐specific hippocampal target genes. We identified *Jdp2* as a likely MR‐specific transcriptional target that is both down‐regulated in fbMRKO mice and up‐regulated in response to restraint stress. Also, *Nos1ap* and *Supv3 l1*, two other genes linked to MR‐specific binding sites, were expressed at a lower levels in fbMRKO mice but did not change upon restraint stress. Classical glucocorticoid target genes *Fkbp5*,* Gilz*,* Per1* and *Sgk1* all responded to restraint stress by increased transcription. Of these targets, only *Fkbp5* showed a substantially lower hippocampal expression in the absence of MR.

Both technical and biological factors could explain the limited success with respect to validating MR‐specific genomic targets. The annotation of binding sites to the nearest gene is not without error because it is possible that another neighbouring gene is affected by the binding locus assessed. We do not have data on spatial chromatin organisation or RNA polymerase activity in the same experimental set‐up, which could enable the proper linking of binding loci to the actual site of transcriptional activity.^29^ To lower the chance of false positive annotations, we focused on binding sites that were located within genes or (proximal) promoter regions. However, even in cases where the putative target is legitimate, we might still have false negative results regarding gene expression changes. Because the hippocampus consists of several subregions and various cell types, we might not be able to detect MR‐dependent regulation that is constrained to a subset of hippocampal cells. Although the ChIP‐seq signal can be sufficiently strong to withstand dilution, gene regulation might be diluted when the average gene expression over the whole hippocampus is assessed because the fold change in hippocampal mRNA expression tends to be modest in response to steroids.^30^ Despite possible false negative results, we were able to find robust changes in several MR‐specific and classical glucocorticoid target genes.

It is of note that gene regulation by MR knockout and restraint stress was validated in a mouse model, whereas the MR/GR binding loci were obtained from experiments in rats. We were able to show MR binding in the mouse hippocampus at five out of six MR targets originally detected in the rat brain. Evolutionary conservation can increase the predictive value of functional GREs.^31^, ^32^ Moreover, because brain MR/GR‐mediated regulation is considered to be part of a general adaptive response, the genes regulated in the rat would also be expected to be affected in mice. However, the species difference is an additional potential cause for absence of mRNA regulation.

The fbMRKO animals become MR deficient during embryonic development and loss of MR protein is completed after birth.^25^ In the present study, down‐regulated MR expression was validated and slight up‐regulation of GR expression in the hippocampus was observed as described previously.^25^ It is possible that MR‐dependent gene expression is normalised as a result of compensation by GR or other factors. We cannot exclude that such compensatory mechanisms might also affect expression of *Jdp2*,* Nos1ap* and *Supv3 l1* in fbMRKO mice. Also, redundancy in gene regulation is not uncommon and, although complete dependence of target genes to a single transcription factor can occur,^33^ it is rare in case of MR and GR signalling. In addition, the binding of nuclear receptors such as MR can have permissive effects on chromatin and could be necessary but not sufficient for transcription. Indeed, as little as 13% of GR binding sites can be linked to transcriptional activity.^34^ Thus, the lack of transcriptional effects might reflect a context dependency.

To begin looking at MR regulation in a relevant context, we chose a restraint stress paradigm in WT mice as a more physiological setting. Mice were stressed in the morning to ensure that basal corticosterone levels were low, and MR activation not necessarily fully maximal.^35^ The classical glucocorticoid target genes all responded in this acute stress situation and, of the MR‐specific targets identified in fbMRKO mice, only *Jdp2* expression was affected. Non‐regulated genes in the restraint stress experiment might still be MR‐dependent but at a lower EC_50_,^36^ or in different contexts, including behavioural paradigms in which fbMRKO animals show changed phenotypes, such as working memory in a radial maze.^25^

For the genes associated with GR‐specific chromatin binding, *Acsl6* and *Mrpl48* showed lower expression levels in fbMRKO mice. In general, the effect size on specifically GR‐associated target gene expression was less pronounced. The fact that these GR targets are down‐regulated and the expression of GR itself is slightly up‐regulated in fbMRKO mice appears to be contradictory. However, this could be a result of indirect effects of MR deficiency. Another explanation is that GR binding takes place at a negative GRE, where GR leads to repression (instead of activation) of the nearby gene.^37^, ^38^

More interestingly, several overlapping targets were down‐regulated in fbMRKO mice: the newly identified *Camk1d* and *Kif1c*, and the classical target *Fkbp5*. This suggests that MR is needed for expression of these genes in the hippocampus. The GR compensatory up‐regulation does not appear to prevent dysregulation of these combined target genes in the absence of MR. It is likely that heterodimerisation of MR and GR is involved in the regulation of overlapping binding sites. *Fkbp5* expression was recently shown to be modulated by MR‐GR heterodimers.^15^ The observation that *Fkbp5* expression is lowered in fbMRKO mice can represent functional consequences of the absence of one of the heterodimerisation partners. Fkbp5 is part of an ultra‐short feedback loop in which it is induced by glucocorticoids, whereas, in turn, Fkbp5 prevents GR activation.^39^ Besides the observed up‐regulation of GR expression itself, the lowered *Fkbp5* levels could contribute to a compensatory mechanism by relieving the repression of GR function to overcome the lack of MR signalling.

Overall, the *Jdp2* gene was the most robust MR target identified in the present study. Initially, Jdp2 was discovered as a negative regulator of activator protein‐1 (AP‐1) function, by dimerising to c‐Jun and preventing transcriptional effects.^40^ Later, it was found that Jdp2 can also act in a stimulating fashion as a coactivator for the progesterone receptor.^41^ In this latter study, Jdp2 was also shown to have a coactivating effect on transactivation by GR, as confirmed by Garza et al.^42^ We found *Jdp2* to be a bona fide MR target. A feedforward mechanism could be speculated in which MR can increase Jdp2 levels, which, in turn, could enhance GR activity. A recent ChIP‐seq study in mouse neuroblastoma cells found the Jdp2 binding motif near both MR‐ and GR‐bound sites.^43^ Besides the differential affinity of MR and GR for their hormone, temporal responses to glucocorticoids could be accounted for by such a feedforward loop. Feedforward models have been described previously for GR^44^ and other nuclear receptors.^45^, ^46^ It is worth noting that Jdp2 has been implicated in AP‐1 modulation during fear extinction,^47^ and polymorphisms in the *Nos1ap* gene have been linked to post‐traumatic stress disorder and depression,^48^ also demonstrating a functional role of these genes in the stress system.

In conclusion, we found three novel hippocampal MR‐specific target genes, comprising *Jdp2*,* Nos1ap* and *Supv3 l1*, of which *Jdp2* is also responsive in an acute stress situation. Dissecting the glucocorticoid response in MR‐specific, common and GR‐specific pathways will enable us to better understand the stress physiology and pathophysiology of stress‐related disorders.

## CONFLICT OF INTERESTS

The authors declare that they have no conflicts of interest.
